# Supplementary material for: Phosphate acts directly on the calcium-sensing receptor to stimulate parathyroid hormone secretion
Source: Nat Commun. 2019 Oct 16;10:4693. doi: 10.1038/s41467-019-12399-9 (PMC6795806; doi:10.1038/s41467-019-12399-9)
Supplement: Supplementary file 1 — Supplementary Information [file 41467_2019_12399_MOESM1_ESM.pdf]

Supplementary Information

"Phosphate acts directly on the Calcium-sensing receptor to stimulate parathyroid hormone secretion"

Centeno *et al.* 2019

Supplementary Figures

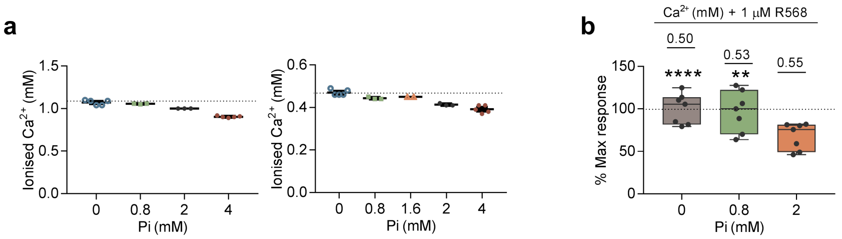

Supplementary Fig. 1: **Measurement of free** Ca^{2+} **in** ***Pi*****-containing buffers. a** The effect of Pi addition on free Ca_o^{2+} concentration was measured in two different buffers, used in *in vitro* experiments (0.5 mM Ca^{2+} and 1μM R568) and in *ex vivo* PTH secretion experiments (with 1.2 mM Ca^{2+} and 1 mg/ml BSA). Increasing Pi concentration in the buffer by +2mM or +4mM decreased the free Ca^{2+} concentration by 10% and 17% respectively. Individual points shown in box-and-whiskers plots, n=2-5 from two independent days. **b** The inhibitory effect of Pi on CaSR-mediated Ca_i^{2+} mobilisation (in 0.5mM Ca^{2+} plus R568) was still observed even when the buffer Ca^{2+} concentration was increased (0.53 and 0.55) to counteract any Pi-mediated reduction in free Ca^{2+}. Data expressed as percent control of the area under the curve for each treatment, with all individual points shown in box-and-whiskers plots, n= 7. Statistical significance was determined using RM-ANOVA with Dunnett's multiple comparisons. **P<0.01 and ****P<0.0001. Source data are provided as a Source Data file.

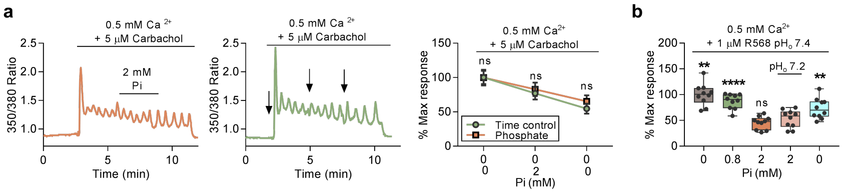

Supplementary Fig. 2: **Inhibitory effect of Pi is CaSR specific and not overcome by acidosis.** **a** Pi does not affect muscarinic receptor-induced Ca_i^{2+}-mobilisation responsiveness in CaSR-HEK cells. Shown are representative Ca_i^{2+} traces (Fura2-ratio) from single cells in response to carbachol in the absence or presence of 2 mM Pi. Changes in Ca_i^{2+}-mobilisation are shown as area under the curve normalised to maximum response. Data are expressed as $mean \pm SEM$; n= 8 from two separate experiments. ns; not significant P>0.05 by paired t-test. **b** The inhibitory effect of Pi on CaSR-induced Ca_i^{2+}-mobilisation is maintained even in the presence of mild acidosis (pH 7.2). Changes in Ca_i^{2+}-mobilisation are shown as area under the curve normalised to maximum response (n= 10, from 3 independent experiments). Data are shown in box-and-whiskers plots. ns, not significant; **P<0.01 and ****P<0.0001 by RM-ANOVA with Dunnett's multiple comparisons test. Source data are provided as a Source Data file.

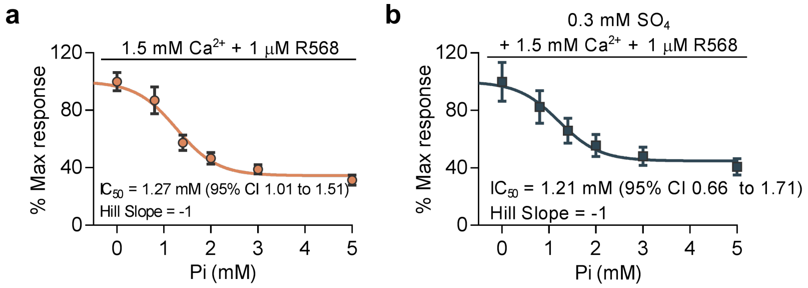

Supplementary Fig. 3: **CaSR is also inhibited by anions in a concentration-dependent manner**. CaSR-mediated Ca_i^{2+}-mobilisation was measured in the presence of increasing concentrations of Pi in the presence of 1.5 mM Ca_o^{2+} and 1μM R568 (**a**), and in the presence of 0.3 mM SO_4 (physiologic) (**b**). Area under the curve was calculated for each treatment and normalised to maximal response. Data were fitted to a four parameter Hill equation (equation 1) for sigmoidal-dose response variable slope. Data fitted best when Hill Slope was constrained to 1, p<0.01 extra sum-of-squares F test. Data expressed as $mean \pm SEM$; n = 7. IC_{50} expressed as mean with 95% confidence intervals. Source data are provided as a Source Data file.

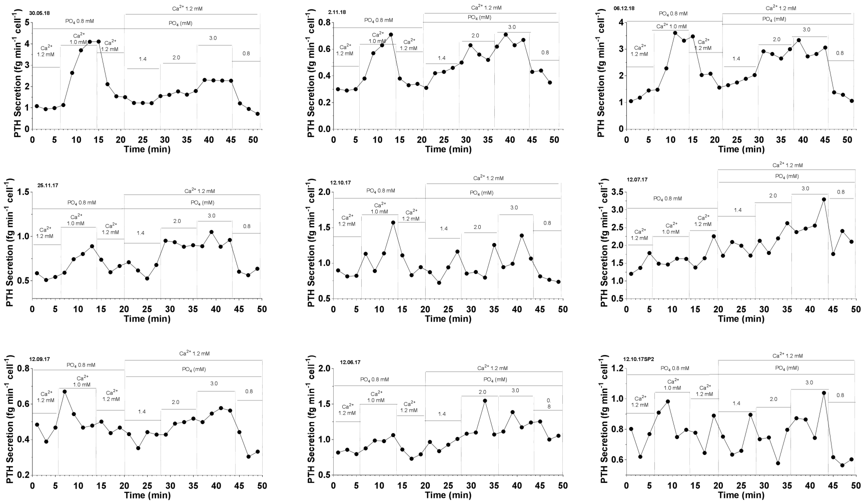

Supplementary Fig. 4: **Pathophysiologic *Pi* concentrations increase PTH secretion in human parathyroid cells.** PTH secretion traces measured every 2 minutes from the individual preparations included in the analysis (N=9) in response to Ca^{2+} and increases in Pi. 1 mM Ca^{2+} was used as internal control to confirm Ca_o^{2+} responsiveness in the cell preparation and CaSR expression. Source data are provided as a Source Data file.

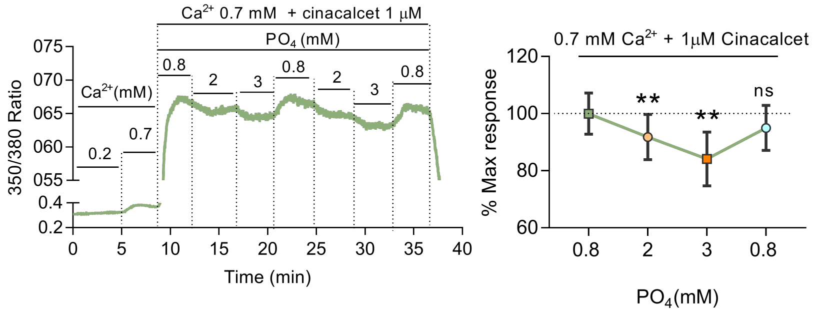

Supplementary Fig. 5: ***Pi* inhibits CaSR-induced Ca_i^{2+} mobilisation in human parathyroid cells.** Representative Ca_i^{2+} trace (Fura2-ratio) showing effect of increasing Pi concentration on a single cell stimulated to induce CaSR-mediated Ca_i^{2+} mobilisation (left). Changes in Ca_i^{2+} are expressed as percentage control of the area under the curve (right). Data are shown as $mean \pm SEM$; n= 9 individual experiments performed on tissue obtained from 5 biologically independent patients. ns; not significant, **P<0.01 by RM-ANOVA with Dunnett's multiple comparisons test. Source data are provided as a Source Data file.

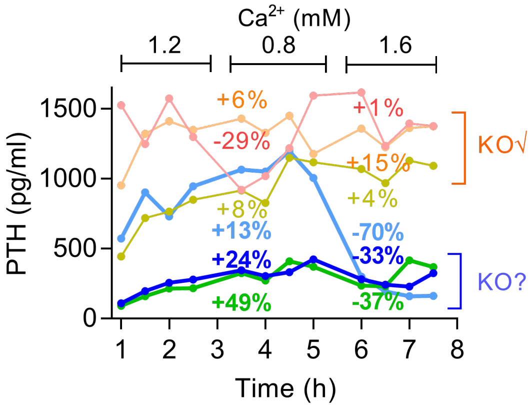

Supplementary Fig. 6: **Individual PTH response profiles from KO *Casr* parathyroid glands.** In the six surviving KO *Casr* mice that exhibited serum PTH concentrations in the 515-1520 μg/ml range, three (shown in warm colors) exhibited no sensitivity to changes in Ca_o^{2+} concentration (i.e. <10% increase in PTH secretion upon exposure to low Ca_o^{2+} concentration and <10% decrease in secretion in high Ca_o^{2+}) and thus were considered as true CaSR knockouts (KO *Casr*). In contrast, the three other mice (shown in cold colors) still exhibited increased PTH secretion *ex vivo* in low Ca_o^{2+} concentration and decreased secretion in high Ca_o^{2+}-containing buffers. Therefore, these mice were excluded from the KO *Casr*. Source data are provided as a Source Data file.

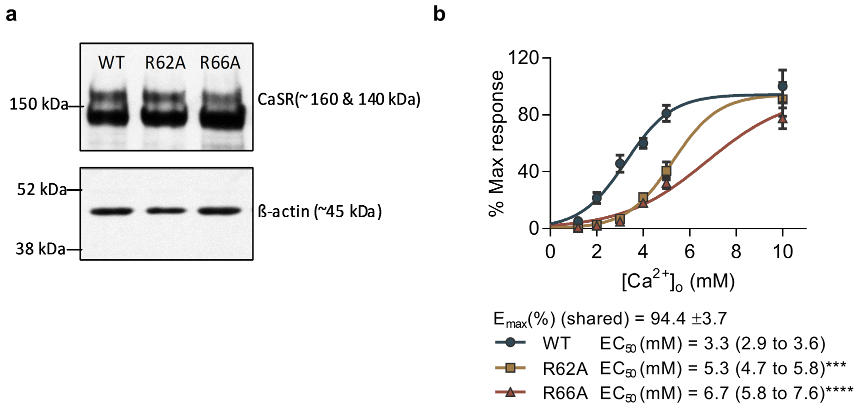

Supplementary Fig. 7: **Characterization of R62A and R66A CaSR mutants**. **a** Immunoblot showing CaSR expression in HEK-293 cells transiently transfected with CaSR^{WT}, CaSR^{R66A} and CaSR^{R62A}, with $\beta - actin$ abundance used as a loading control. **b** Ca_o^{2+} concentration-dependence curves showing Ca_i^{2+}-mobilisation in HEK-293 cells transiently expressing CaSR^{WT}, CaSR^{R66A} and CaSR^{R62A}. Data expressed as area under the curve normalised to maximal response and fitted to a four parameter Hill equation (equation 1) for sigmoidal-dose response variable slope. Data fitted best when E_{max}, expressed as $\% \; mean \pm SEM$, was shared among data sets, p<0.01 extra sum-of-squares F test (n = 8-10 from three independent transfections). EC_{50}, expressed as mean (95% confidence interval). Data analyzed using RM-ANOVA, Dunnett's multiple comparisons ***P<0.001. Source data are provided as a Source Data file.

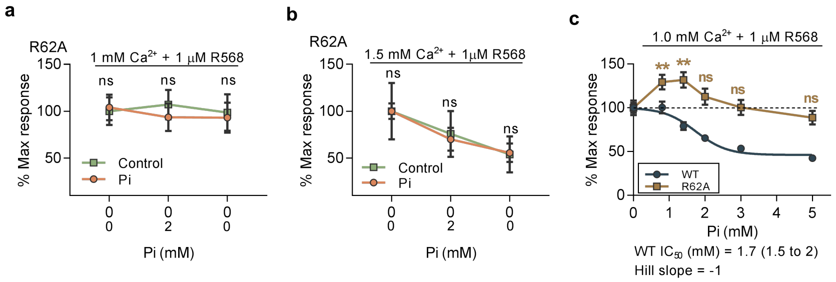

Supplementary Fig. 8: ***$CaSR^{R62A}$* is not inhibited by *Pi***. CaSR^{R62A}-induced Ca_i^{2+} mobilisation in response to either 1 mM Ca_o^{2+} (plus R568) (**a**), or 1.5 mM Ca_o^{2+} (plus R568) (**b**) in the absence and then presence of 2 mM Pi. Fura-2 ratio changes expressed as area under the curve normalised to maximal response, n=9 and 10 (**a**) and n=5 and 7 (**b**). **c** Pi concentration-effect curves on Ca_i^{2+}-mobilisation upon stimulation with 1 mM Ca_o^{2+} and R568 for CaSR^{WT} and CaSR^{R62A}. CaSR^{WT} was inhibited in a concentration-dependent manner and data fitted to a four parameter Hill equation (equation 1) for sigmoidal-concentration dose response variable slope, whereas CaSR^{R62A} was not inhibited by Pi and did not fit to the equation (n= 11 (CaSR^{WT}) and n=10 (CaSR^{R62A}), from three independent experiments). Data expressed as area under the curve normalised to maximal response (% $mean \pm SEM$), EC_{50}, expressed as mean (95% confidence interval) and E_{max} expressed as $\%mean \pm SEM$. CaSR^{R62A} data was analyzed using RM-ANOVA, Dunnett's multiple comparisons. ns, not significant; **P<0.01. Source data are provided as a Source Data file.

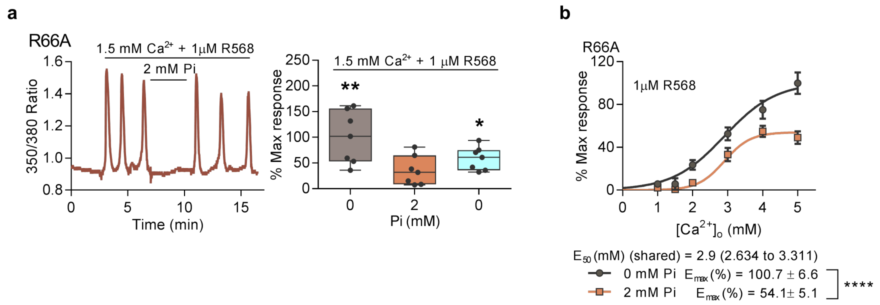

Supplementary Fig. 9: $CaSR^{R66A}$ **is inhibited by *Pi*.** **a** CaSR^{R66A}-induced Ca_i^{2+}-mobilisation in response to 1.5 mM Ca_o^{2+} (plus R568) is inhibited by increasing Pi concentration. A representative Fura-2 ratio trace from a single cell is shown (left) with data reported as percentage control of the area under the curve (right); n= 7, from 3 independent experiments. Data shown in box-and-whiskers plots. **b** Ca_o^{2+} concentration-effect curves for Ca_i^{2+}-mobilisation in the presence and absence of Pi in cells transiently expressing CaSR^{R66A} (n = 8/10, from three independent transfections). Data expressed as area under the curve normalised to maximal response ($\%\ mean \pm SEM$) and fitted to a four parameter Hill equation (equation 1) for sigmoidal-dose response variable slope. EC_{50}, expressed as mean (95% confidence interval) and E_{max} expressed as $\%mean \pm SEM$. Data expressed as $\%\ mean \pm SEM$, and analyzed using RM-ANOVA, Dunnett's multiple comparisons (**a**) or unpaired t-test (**b**). *P<0.05, **P<0.01,****P<0.0001. Source data are provided as a Source Data file.
