# Supplementary material for: Phosphate acts directly on the calcium-sensing receptor to stimulate parathyroid hormone secretion
Source: Nat Commun. 2019 Oct 16;10:4693. doi: 10.1038/s41467-019-12399-9 (PMC6795806; doi:10.1038/s41467-019-12399-9)
Supplement: Supplementary file 3 — Description of Additional Supplementary Files [file 41467_2019_12399_MOESM3_ESM.pdf]

Description of Additional Supplementary Files

File name: Supplementary Movie 1

Description: CaSR structure animation from active (closed) to inactive (open) conformation, highlighting the R62-E277 salt bridge (pink sticks) and R66-S301 hydrogen bond (green sticks). Phosphate ions are shown as red spheres and calcium ions shown as green spheres (animation created using Pymol).
