# Supplementary material for: Phosphate acts directly on the calcium-sensing receptor to stimulate parathyroid hormone secretion
Source: Nat Commun. 2019 Oct 16;10:4693. doi: 10.1038/s41467-019-12399-9 (PMC6795806; doi:10.1038/s41467-019-12399-9)
Supplement: Supplementary file 5 — Reporting Summary [file 41467_2019_12399_MOESM5_ESM.pdf]

nature research

Corresponding author(s): Donald T. Ward

Last updated by author(s): 26/07/2019

Reporting Summary

Nature Research wishes to improve the reproducibility of the work that we publish. This form provides structure for consistency and transparency in reporting. For further information on Nature Research policies, see Authors & Referees and the Editorial Policy Checklist.

Statistics

For all statistical analyses, confirm that the following items are present in the figure legend, table legend, main text, or Methods section.

n/a	Confirmed	
☐	☒	The exact sample size (*n*) for each experimental group/condition, given as a discrete number and unit of measurement
☐	☒	A statement on whether measurements were taken from distinct samples or whether the same sample was measured repeatedly
☐	☒	The statistical test(s) used AND whether they are one- or two-sided<br>*Only common tests should be described solely by name; describe more complex techniques in the Methods section.*
☐	☒	A description of all covariates tested
☐	☒	A description of any assumptions or corrections, such as tests of normality and adjustment for multiple comparisons
☐	☒	A full description of the statistical parameters including central tendency (e.g. means) or other basic estimates (e.g. regression coefficient) AND variation (e.g. standard deviation) or associated estimates of uncertainty (e.g. confidence intervals)
☐	☒	For null hypothesis testing, the test statistic (e.g. *F*, *t*, *r*) with confidence intervals, effect sizes, degrees of freedom and *P* value noted<br>*Give P values as exact values whenever suitable.*
☒	☐	For Bayesian analysis, information on the choice of priors and Markov chain Monte Carlo settings
☒	☐	For hierarchical and complex designs, identification of the appropriate level for tests and full reporting of outcomes
☒	☐	Estimates of effect sizes (e.g. Cohen's *d*, Pearson's *r*), indicating how they were calculated

Our web collection on statistics for biologists contains articles on many of the points above.

Software and code

Policy information about availability of computer code

Data collection	Intracellular calcium mobilisation data were collected using MetaFluor® Fluorescence Ratio Imaging Software.<br>ERK phosphorylation and CaSR Western Blots were developed using Chemi-Doc® Image Lab 6.0 (BIO-RAD)
Data analysis	R package "PKNCA Perform Pharmacokinetic Non-Compartmental Analysis" was used to calculate area under the curve for calcium mobilisation experiments. PyMOL 2.07 was used to visualize crystal structures and to perform in silico analysis of the CaSR-ECD and phosphate interactions. R package "Seqinr: Biological Sequences Retrieval and Analysis", MEGA 7 and WebLogo 2.8.2 were used to analyze phosphate binding sites conservation across species. Graphpad Prism v7 was used to generate figures and to perform statistical analysis. MetaFluor® Fluorescence Ratio Imaging was used to calculate Fura ratio 350/380. Image Lab 6.0 (Bio-Rad) was used to quantified band intensity in Western Blots.

For manuscripts utilizing custom algorithms or software that are central to the research but not yet described in published literature, software must be made available to editors/reviewers. We strongly encourage code deposition in a community repository (e.g. GitHub). See the Nature Research guidelines for submitting code & software for further information.

Data

Policy information about availability of data

All manuscripts must include a data availability statement. This statement should provide the following information, where applicable:

- Accession codes, unique identifiers, or web links for publicly available datasets
- A list of figures that have associated raw data
- A description of any restrictions on data availability

All data generated during this study are included in this paper and its figures and supplementary information. In addition, publicly available data were analyzed in this study and are cited wherever relevant.

Field-specific reporting

Please select the one below that is the best fit for your research. If you are not sure, read the appropriate sections before making your selection.

☒ Life sciences ☐ Behavioural & social sciences ☐ Ecological, evolutionary & environmental sciences

For a reference copy of the document with all sections, see nature.com/documents/nr-reporting-summary-flat.pdf

Life sciences study design

All studies must disclose on these points even when the disclosure is negative.

Sample size	Sample sizes were not predetermined by statistical methods. For human and murine parathyroid tissue, samples size were determined by availability of tissue and taking into account local ethical and reductionist animal use. For in vitro experiments we followed standard practices in the field, this consists of at least 3 independent experiments performed in triplicates. We believe this to be sufficient, and our estimates of the mean and SEM did not deviate by incorporating more experiments. The sample size for each experiment have been listed in the figure legends.
Data exclusions	For murine PTH secretion, data were filter prior to statistical analysis to remove PT glands from the CaSR knock-out group with CaSR expression (incomplete Cre recombination). This was evaluated by two criteria; a) Low serum PTH levels (<10% higher than littermates controls) and b) Calcium responsiveness (>10% PTH increase/decrease) (Supplementary Figure 5).
Replication	All attempts at replication succeed. In general, our data shows mean +/- SEM from 3-5 independent experiments (biological replicates) performed in triplicates (technical replicates of the biological replicates). All figures show individual data points.
Randomization	Randomization was not attempted or necessary for this study.
Blinding	Blinding was not attempted or needed.

Reporting for specific materials, systems and methods

We require information from authors about some types of materials, experimental systems and methods used in many studies. Here, indicate whether each material, system or method listed is relevant to your study. If you are not sure if a list item applies to your research, read the appropriate section before selecting a response.

Materials & experimental systems

n/a	Involved in the study	
☐	☒	Antibodies
☐	☒	Eukaryotic cell lines
☒	☐	Palaeontology
☐	☒	Animals and other organisms
☐	☒	Human research participants
☒	☐	Clinical data

Methods

n/a	Involved in the study	
☒	☐	ChIP-seq
☒	☐	Flow cytometry
☒	☐	MRI-based neuroimaging

Antibodies

Antibodies used	All antibodies were commercially available. Primary antibodies were: Phospho-p44/42 MAPK (Erk1/2) (Thr202/Tyr204) (E10) Mouse (1:3000 dilution) from Cell Signalling (Cat. number 9106), Calcium Sensing Receptor monoclonal mouse antibody (5C10, ADD) (1:3000 dilution) from Thermo Fisher Scientific (Cat. number MA1-934), and β-actin-Peroxidase monoclonal (AC-15) mouse antibody (1:25.000 dilution) from MERCK (Cat. number A3854). Secondary antibody was horseradish peroxidase-conjugated horse anti-mouse IgG (1:10.000) from Cell Signalling (Cat. number 7076).
Validation	These antibodies were validated by the vendor and published studies.

Eukaryotic cell lines

Policy information about cell lines

Cell line source(s)	We used human embryonic kidney cells 293, commercially available [HEK-293] (ATCC® CRL-1573), stably expressing the CaSR wild-type or R62A mutant, and transiently expressing the R66A mutant.
Authentication	Cell lines are maintained by the supplier. Additional Western blots were performed by the authors of this study to confirm CaSR expression (Supplementary Figure 6).

Mycoplasma contamination | All cell lines were tested and are free of mycoplasma contamination.

Commonly misidentified lines (See ICLAC register) | No commonly misidentify cell lines were used.

Animals and other organisms

Policy information about studies involving animals; ARRIVE guidelines recommended for reporting animal research

Laboratory animals

Parathyroid glands from male and female 7-10 day-old with C57 background control mice and homozygous parathyroid gland-specific deletion of exon 7 (encoding the CaSR transmembrane and intracellular domains, and loops) of the CaSR (KO CaSR) mice were used in the study. KO CaSR were generated by breeding CaSR flox/flox mice with transgenic mice expressing Cre-recombinase under the control of the PTH promoter for parathyroid glands (PTH-Cre, The Jackson Laboratory). Control mice were littermates that did not express both transgene simultaneously. Mice were housed in the animal facilities of the University of San Francisco California Veterans Affairs Medical Center and were maintained under normal mouse chow.

Wild animals

No wild animals were involved in the study

Field-collected samples

No field-collected samples were used in this study

Ethics oversight

Institutional Animal Care and Use Committee (IACUC) at San Francisco Veterans Affairs Medical Center approved the study protocol (Protocol 18-017).

Note that full information on the approval of the study protocol must also be provided in the manuscript.

Human research participants

Policy information about studies involving human research participants

Population characteristics

Normal human parathyroid tissue was obtained from neck surgeries performed on male and female, aged >18 years, of all ethnicities at the Royal North Shore Hospital (St Leonards, NSW Australia) and the Mater Misericordiae Hospital (North Sydney, NSW).

Recruitment

Male and female patients undergoing neck surgery and involving thyroidectomy were informed about the possibility of using the removed tissue for experimental purposes. All patients involved in the study provided written informed consent for the use of tissue for experimental purposes.

Ethics oversight

Approval for access to human parathyroid tissue was provided by the Human Research Ethics Committee of St Vincents Health Network, Darlinghurst, NSW 2010 (SVH File Number 11/067).

Note that full information on the approval of the study protocol must also be provided in the manuscript.
